# High-Frequency Repetitive Transcranial Magnetic Stimulation (rTMS) in the First Episode of Catatonia: A Series of Four Cases

**DOI:** 10.7759/cureus.49112

**Published:** 2023-11-20

**Authors:** Abid Rizvi, Faisal Shaan, Karrar Husain, Mohammed Reyazuddin, Nayab Anjum

**Affiliations:** 1 Psychiatry and Behavioral Sciences, West Virginia University, Morgantown, USA; 2 Psychiatry, Jawaharlal Nehru Medical College, Aligarh, IND; 3 Psychiatry, Texas Tech University Health Sciences Center, Lubbock, USA

**Keywords:** brain stimulation, mood disorder, schizophrenia, rtms, catatonia

## Abstract

Four patients with benzodiazepine non-responsive catatonia were administered repetitive transcranial magnetic stimulation (rTMS) at the left dorsolateral prefrontal cortex at 120% of resting motor threshold, frequency of 10Hz, with a total of 3,000 pulses/session. Patients with mood disorders showed good responses. One patient with chronic resistant schizophrenia had worsening catatonic symptoms during rTMS that responded to electroconvulsive therapy. Maximum response was observed between sessions 8 and 12.

## Introduction

Catatonia, characterized by distinctive psychomotor abnormalities, is categorized into two primary forms: retarded and excited. The retarded form encompasses symptoms, including posturing, grimacing, negativism, waxing flexibility, echolalia, echopraxia, stereotypies, verbigeration, and automatic obedience. Conversely, excited catatonia, sometimes referred to as malignant catatonia, has acute psychomotor agitation that can be accompanied by hyperthermia and autonomic dysfunction. This latter form demands prompt intervention to avert rapid deterioration and death. The incidence of catatonic symptoms in acute psychiatric settings is estimated to range between 9% and 17%, with the retarded form being more prevalent compared to the excited form [1,2].

In the absence of treatment, catatonia can be fatal. The efficacy of lorazepam in managing catatonia suggests a dysregulation in the Gamma-aminobutyric acid (GABA) neurotransmitter systems. This hypothesis is further supported by the observed decrease in GABA-A receptor density in patients with catatonia [3-5].

The primary treatment modalities for catatonia, as recommended by clinical guidelines, are benzodiazepines and electroconvulsive therapy (ECT) in patients who do not respond to benzodiazepines [6]. However, ECT is often constrained by limited availability, stigma, perceived risks, and anesthesia requirements [7]. Consequently, non-invasive brain stimulation techniques like repetitive Transcranial Magnetic Stimulation (rTMS) and transcranial direct current stimulation (tDCS), which modulate neuronal activity, have emerged as promising alternatives. These techniques are essential in cases where traditional treatments are either ineffective or inaccessible. Patients with catatonia have shown benefits from excitability-enhancing rTMS and tDCS, mainly targeting the dorsolateral prefrontal cortex (DLPFC), which has been implicated in its pathophysiology [3,4,7].

Earlier reports of improvement in catatonic symptoms through rTMS primarily emanate from case studies. Our research contributes to this body of knowledge by presenting case series of four patients with catatonia, exploring potential predictors of response, and laying the groundwork for future research. This study aims to bridge the knowledge gap in optimizing rTMS protocols and predicting responses in the treatment of catatonia. Ethical approval for this study was granted by the institutional review board (reference number IECJNMC/945). Informed consent was duly obtained from the patient or the patient’s surrogate decision-makers/guardians. In cases where the patient could communicate, their assent was also secured. Additionally, informed consent was sought before each rTMS session from the patient’s guardian or immediate family, and the treatment was discontinued upon observing no further improvement or upon withdrawal of consent by the patient’s surrogate decision-makers.

## Case presentation

Case 1

A 16-year-old male from India presented to the psychiatric outpatient department of a tertiary care hospital, exhibiting symptoms of catatonia that had lasted for 25 days. The patient had diminished verbal communication that had progressed to complete mutism. He displayed a vacant stare, held sustained postures, demonstrated rigidity, and had a markedly reduced oral intake.

The patient was diagnosed with schizophrenia at the age of 13. Despite undergoing various pharmacological interventions, symptom resolution was not attained until the initiation of clozapine therapy. With the introduction of clozapine, the patient’s psychiatric symptoms were well-managed, allowing him to return to school and lead a functional life. At the time of his presentation to the hospital, he had been on a stable 200 mg daily dose of clozapine before the onset of catatonic symptoms. Due to financial constraints and lack of availability, serum clozapine levels were not assessed.

For diagnostic evaluation, a series of tests were conducted, including a Complete Blood Count (CBC), Comprehensive Metabolic Panel (CMP), Thyroid Function Tests (TFT), urine drug screening, and brain magnetic resonance imaging (MRI). All tests yielded normal results. The patient was subsequently diagnosed with catatonia according to the Diagnostic and Statistical Manual of Mental Disorders, Fifth Edition (DSM-5) criteria, with a Bush-Francis Catatonia Rating Scale (BFCRS) score of 18.

BFCRS is a comprehensive 23-item clinician-rated tool designed for diagnosing catatonia. The first 14 items are intended for initial screening and cover a wide range of common catatonia symptoms such as immobility, mutism, and staring, as well as classical signs like waxy flexibility and echo phenomena. The diagnosis of catatonia is confirmed when at least two symptoms are present for more than 24 hours. Each symptom is rated on a scale from 0 to 3, with 0 indicating absence and 3 indicating severe presence, to assess the severity of catatonic features. The scale is known for its high reliability, ensuring consistent assessments across different clinical scenarios. It is not only instrumental in diagnosing catatonia but also valuable in monitoring the response to treatment in clinical settings.

Given the diagnosis, treatment with lorazepam was initiated. Lorazepam was started at 2 mg on the first day; the dose was escalated to 10 mg/day in divided doses by the third day. Concurrently, clozapine therapy was maintained, following literature suggesting that abrupt discontinuation could exacerbate catatonic symptoms. With lorazepam therapy, there is no improvement in catatonic symptoms. The patient had excessive sedation, because of which any further increase was not attempted.

Upon discussing further treatment options with the patient’s guardian, initial consent for ECT was declined. However, consent was granted for rTMS. The patient underwent rTMS targeting the left DLPFC (L-DLPFC) using the MediStim (MS-30) machine with figure-of-8 coils. F3 of the 10-20 EEG system was used to locate the left DLPFC. The sessions were conducted five days a week, each lasting 37.5 minutes and utilizing parameters that included 120% of the resting motor threshold, a frequency of 10 Hz, and a total of 3,000 pulses per session. The patient’s guardian provided informed consent before each rTMS session, and the BFCRS was used to monitor symptom severity. Unfortunately, after 10 sessions of rTMS, there was a worsening of the patient’s catatonic symptoms, reflected in a BFCRS score that increased to 22. At this juncture, the patient’s guardian withdrew consent for continued rTMS and approved ECT. Following five sessions of ECT, the patient demonstrated a favorable response, resolving his catatonic state (Table 1). He was subsequently discharged, continuing on his established regimen of 200 mg of clozapine.

**Table 1:** Characteristics of patients receiving rTMS and improvement in BPCRS score BFCRS - Bush-Francis Catatonia Rating Scale; rTMS - repetitive transcranial magnetic stimulation

		Case 1	Case 2	Case 3	Case 4
Age (years)/sex		16/male	18/female	20/male	28/female
Duration of catatonic symptoms		25 days	8 days	One month	One week
Underlying Psychiatric illness		Resistant schizophrenia	Bipolar disorder in depressive phase	schizophrenia	Severe MDD (post-partum onset)
Age of diagnosis of psychiatric illness (years)		13	17	16	28
Dose of lorazepam tried Before rTMS		10mg/day	8mg/day	12mg/day	8mg/day
Non-benzodiazepine psychotropic taken before rTMS		Clozapine 200mg /day	Lithium 900mg/day Quetiapine 300 mg /day	Olanzapine 20 mg /day	Fluoxetine 20 mg /day
Medication continued during rTMS		Clozapine 200mg /day, Lithium 900mg/day	none	Fluoxetine 20 mg /day
Number of rTMS sessions given		10	16	14	15
BFCRS Score After rTMS sessions	Baseline	18	16	18	20
	4 sessions	18	16	18	15
	8 sessions	19	14	16	14
	12 sessions	Not applicable	10	13	7
	Final sessions	22	8	13	7

Case 2

An 18-year-old female patient was admitted to the Emergency Department of a tertiary care hospital in India, exhibiting symptoms indicative of catatonia. For the past eight days, the patient had developed reduced oral intake, posturing, and decreased speech output, eventually progressing to complete mutism. The patient had a history of bipolar disorder, diagnosed at the age of 17. Initially, her symptoms had responded well to a regimen of Quetiapine 300 mg/day. Due to two more manic episodes within the past year, Lithium was added to her medication plan, titrated up to 900 mg/day. She had remained asymptomatic for about six months on this regimen but had discontinued her medications and resumed them only a week before her current hospital admission.

Family history revealed no instances of psychiatric illnesses, and the patient had no other medical comorbidities. Diagnostic assessments yielded unremarkable results, including CBC, CMP, TFT, urine drug screening, pregnancy test, and brain MRI. According to the DSM-5, the patient was diagnosed with catatonia. Her BFCRS score was 16 at baseline.

Therapeutic intervention was initiated immediately. Quetiapine was discontinued, while Lithium was continued during the treatment of catatonia. Lorazepam was started at a dose of 4 mg/day on the first day and increased to 8 mg/day by the second day in divided doses. Despite the increase, there was no observable therapeutic response. The patient became excessively somnolent, precluding further dose escalation and necessitating the insertion of a nasogastric (NG) tube for nutritional support. ECT was considered but declined by the patient's guardian, who consented to rTMS as an alternative.

The patient received rTMS focused on the left DLPFC employing the MediStim (MS-30) apparatus, which utilizes figure-of-8 coils. The F3 position in the 10-20 EEG system was the reference point for targeting the left DLPFC. Treatment sessions were scheduled five times weekly, with each session lasting for 37.5 minutes. The operational parameters for these sessions included a magnetic field intensity set at 120% of the patient's resting motor threshold, a frequency of 10 Hz, and a delivery of 3,000 magnetic pulses in each session. Informed consent was obtained from the patient's guardian before each session. The BFCRS was used to gauge symptom severity at baseline and after each rTMS session, revealing a decrease from a score of 16 at baseline to 8 by the end of the 16th session.

Post-treatment, the patient showed a significant clinical improvement in her catatonia symptoms, enabling her to engage in most activities of daily living. She did, however, continue to experience depressive symptoms. As a result, quetiapine was reinitiated at a dose of 100 mg and titrated up to 300 mg at bedtime. She was then discharged and followed up as an outpatient. Within three weeks, her depressive symptoms had improved considerably. Over a one-year follow-up period, the patient maintained her functional levels and did not experience a recurrence of her catatonic symptoms.

Case 3

A 20-year-old Indian male, previously diagnosed with schizophrenia at age 16, presented to the hospital with symptoms of decreased oral intake, rigidity, echolalia, mannerisms, episodic psychomotor agitation, and posturing that had been evolving over a month. A day before admission, his oral and fluid intake had ceased altogether, leading to dehydration and necessitating the initiation of intravenous fluids. The patient had a blood pressure of 90/50 mmHg and tachycardia with a pulse rate of 110 bpm; however, he was afebrile.

Despite a comprehensive battery of diagnostic tests - CBC, CMP, TFT, urine drug screening, cerebrospinal fluid examination, and brain MRI - no remarkable findings were observed. Serum creatine kinase levels also remained within the normal range. At the time of presentation, the patient had been on a stable regimen of olanzapine 20 mg/day, and there was no identifiable precipitating factor for his catatonic symptoms, such as worsening psychosis or non-compliance with medication.

Based on DSM-5 criteria and a BFCRS score of 18, the diagnosis of catatonia was established. Olanzapine was discontinued, and the patient was started on lorazepam. Despite an escalation from an initial 4 mg/day dose to 12 mg/day in divided dosage by day three, the patient showed no noticeable response after four days of lorazepam treatment.

After discussing treatment, the patient’s father, who also served as his healthcare surrogate, declined consent for ECT. However, permission was granted to proceed with rTMS.

The treatment involved the patient receiving repetitive rTMS aimed at the left DLPFC, facilitated by the MediStim (MS-30) device equipped with figure-of-8 coils. To accurately target the left DLPFC, the F3 landmark from the 10-20 EEG system was employed. These rTMS sessions were administered five times per week, lasting 37.5 minutes. The settings for the sessions included an intensity of 120% of the patient's resting motor threshold, a pulse frequency of 10 Hz, and the administration of 3,000 pulses is performed in each session.

Despite the rigorous protocol, only a marginal improvement was observed after 12 sessions, with a decrease in the BFCRS score from 18 to 13 (Table 1). Subsequent sessions did not result in further improvement. The collective decision was made to discontinue rTMS treatment. The patient showed some improvement in symptoms like oral intake, rigidity, and echolalia. He was subsequently discharged from the hospital but was unfortunately lost to follow-up, leaving his longer-term outcome uncertain.

Case 4

A 28-year-old female, in the third week post-partum after a cesarean section for her third childbirth, arrived at the emergency department. She exhibited increasingly severe catatonic symptoms, which had been progressively worsening over the previous week. Initially, these symptoms manifested as prolonged periods of standing and sitting in the same posture but quickly escalated to complete mutism and stupor within just one week. She was diagnosed with post-partum depression just three days following her recent delivery. At that time, she exhibited melancholic mood, reduced sleep, and decreased appetite, compounded by episodic panic attacks. She also reported overwhelming feelings of helplessness and hopelessness, leading her to relinquish the care of her newborn to her mother. Despite being prescribed fluoxetine 20 mg daily by her obstetrician-gynecologist, she saw no improvement after 10 days of treatment.

Upon arrival at the emergency room, her vital signs were stable, and she was afebrile. Comprehensive diagnostic tests were conducted, including CBC, CMP, TFT, urine drug screening, and MRI. All test results were unremarkable, revealing no significant abnormalities. Serum creatine kinase levels were also within the normal range. Notably, both her past medical history and family history were devoid of psychiatric illnesses. She had, however, managed gestational diabetes mellitus with insulin during her pregnancies and underwent cesarean section deliveries for all her three children, but with no previous instances of post-partum depression.

She was diagnosed with both post-partum depression and catatonia as per DSM-5. Treatment with lorazepam was initiated, starting at 2 mg/day and gradually titrating the dose to 8 mg/day in divided dosage. Meanwhile, fluoxetine was continued. Unfortunately, apart from sedation, the patient showed no significant response to the treatment after three days. ECT was considered but ultimately declined by her husband, who acted as her healthcare surrogate. Alternatively, consent was secured for rTMS, prompting the discontinuation of lorazepam. Her BFCRS score was initially 20, indicating severe catatonia.

The patient received rTMS treatments focused on the left DLPFC using the MediStim (MS-30) equipment, which features figure-of-8 coils. The F3 site of the 10-20 EEG layout was utilized to pinpoint the left DLPFC. These rTMS sessions were conducted five times a week, each lasting 37.5 minutes. The treatment parameters consisted of a magnetic field strength set at 120% of the patient's resting motor threshold, a 10 Hz frequency, and 3000 magnetic pulses per session.

She began improving after just four rTMS sessions, as evidenced by a decrease in her BFCRS score to 15. The most significant therapeutic effect was observed between the eighth and 12th sessions, with her BFCRS score decreasing to 7 (Table 1). Despite administering a total of 15 rTMS sessions, no additional improvement was seen beyond the 12th session, leading to the discontinuation of rTMS. However, her depressive symptoms markedly improved, enabling her to resume the care of her newborn. She continued her fluoxetine treatment, and at a three-week outpatient follow-up, she returned to her baseline level of functioning.

## Discussion

Our study engaged four patients with unique clinical presentations but a common absence of organic etiology for their catatonia. It is worth noting that all four patients were experiencing catatonia for the first time and reported no adverse effects during the rTMS treatments.

Regarding therapeutic responses, patient 1, suffering from treatment-resistant schizophrenia, found no relief from catatonic symptoms under the rTMS regimen. After ten sessions, the guardian favored ECT, to which the patient responded favorably. Patients 2 and 4, who were diagnosed with different types of mood disorders, displayed a substantial reduction-exceeding 50%-in their Bush-Francis Catatonia Rating Scale (BFCRS) scores. Meanwhile, patient 3 achieved only partial alleviation of symptoms, with no change in the BFCRS score after completing 12 sessions of rTMS.

Patient 4, who had been struggling with severe post-partum depression and concomitant catatonia, showed the most pronounced response to treatment, evident by an approximately 65% reduction in BFCRS scores. This finding significantly enriches the existing medical literature, given its novelty.

Comparative analysis with existing studies highlights a variance in treatment outcomes. Existing literature has reported significant improvements in 11 out of 12 cases [8]. However, only half of the patients in our study reached this milestone. This divergence may be attributed to publication bias [9]. Moreover, our findings reiterate that mood disorders associated with catatonia generally offer a more favorable prognosis compared to schizophrenia [4].

Our rTMS protocol explicitly focused on the left DLPFC, adhering to methodologies validated by earlier research [8,10,11]. Stimulatory treatments targeting the left DLPFC, particularly high-frequency and intermittent theta burst stimulation, were frequently used and showed positive results in treating catatonia in earlier studies [8]. Our study contributes to this line of investigation by strengthening the existing data.

Two particular findings warrant special attention. The first is the observed trend that maximum therapeutic benefits -quantified by BFCRS scores - typically manifest between the eighth and 12th sessions. Earlier case reports partially corroborated this finding, in which the patient showed improvement in 7-10 sessions [12,13]. Consequently, further controlled studies are essential to affirm the optimal number of treatment sessions. Secondly, the marked improvement in patient 4 brings into focus the potential of rTMS in treating cases of post-partum depression associated with catatonia, which has not been documented before.

This study on rTMS for catatonia showcases several strengths, such as including patients with varied psychiatric conditions like schizophrenia, bipolar disorder, and post-partum depression. The use of DSM-5 criteria and the Bush-Francis Catatonia Rating Scale ensures a methodical and reproducible approach. Detailed rTMS protocols and adherence to ethical standards, including informed consent, add to the study's credibility. Its real-world tertiary care setting also broadens the applicability of the findings.

Conversely, the study faces limitations, including a small sample size, which may affect the generalizability of its results. The lack of a control group limits the ability to attribute improvements to rTMS definitively. A short follow-up period raises questions about long-term effects, while concurrent medication use could have impacted the results, obscuring rTMS's sole effect. Subjective assessment scales and the non-blinded design might introduce bias. The specific geographic and clinical context also limits broader applicability. The study does not delve into the mechanisms of rTMS on catatonia, leaving a gap in understanding its efficacy. Despite offering valuable insights, these limitations should be weighed in interpreting the study's implications for clinical practice and future research.

## Conclusions

In conclusion, our study of four patients with catatonia of differing psychiatric origins reveals mixed efficacy of rTMS. Notably, patients with mood disorders responded more favorably than those with schizophrenia, as gauged by the BFCRS. Two key observations emerged: maximal therapeutic gains occur between the eighth and 12th rTMS sessions, and rTMS may be particularly effective for catatonia in post-partum depression, as evidenced by patient 4. Our study diverges from existing literature, possibly due to publication bias and differences in underlying psychiatric conditions. The limitations of this case series, such as its small sample size and inability to rule out other treatments' effects, necessitate further, more robust trials. Future research should explore variations in rTMS protocols and target-specific conditions like post-partum depression with catatonia.
